# Renewable Lignocellulosic and Starch Feedstocks for Biodegradable Polymers in Kazakhstan: Resource Chemistry, Conversion Routes, and Implementation Priorities

**DOI:** 10.3390/polym18202493

**Published:** 2026-10-12

**Authors:** Aisha Nurlybayeva, Ayazhan Yegissinova, Elmurod Egamberdiev, Nurbanu Sarova, Zulfiya Unerbayeva, Raikhan Rakhmetullayeva, Dina Asylbekova, Nazgul Murzakasymova, Damen Nurgaliyeva, Nurana Kerimova, Bakytgul Kussainova

**Affiliations:** 1 Department of Chemistry and Chemical Technology, M. Kh. Dulaty Taraz University, Taraz 080000, Kazakhstan; rustem_ergali@mail.ru (A.N.); naz1282@mail.ru (N.M.); 2 Faculty of Chemistry, Tashkent State Technical University, University Str. 2, Tashkent City 100095, Uzbekistan; el.0919@mail.ru; 3 Department of Chemistry, Faculty of Natural Sciences and Geography, Kazakh National Pedagogical University Named After Abai, Almaty 050010, Kazakhstan; n.sarova@abaiuniversity.edu.kz; 4 Department of Chemistry, Kazakh National Pedagogical University Named After Abai, Almaty 050010, Kazakhstan; uner_68@mail.ru; 5 Farabi University, Almaty 050040, Kazakhstan; raikhan.rakhmetullayeva@gmail.com; 6 Mukhtar Auezov South Kazakhstan Research University, Shymkent 160012, Kazakhstan; 7 Astana International University, Astana 010000, Kazakhstan; nurgalieva0109@bk.ru; 8 School-Lyceum No. 35 Named After Nazir Torekulov, Astana 010000, Kazakhstan; kerimova.nurana.02@mail.ru; 9 M. Utemisov West Kazakhstan University, Uraslsk 090000, Kazakhstan; nurbol-bakytgul@mail.ru

**Keywords:** Kazakhstan, lignocellulose, starch, biodegradable polymers, thermoplastic starch, cellulose, nanocellulose, lignin, poly(lactic acid), polyhydroxyalkanoates, circular bioeconomy

## Abstract

Kazakhstan combines a large agricultural-residue base with an expanding grain-processing sector, creating a credible platform for biodegradable polymers if feedstock availability is matched to polymer chemistry, pretreatment intensity, and realistic end-of-life routes. This critical review integrates Kazakhstan-specific production data with the chemistry of starch, cellulose, hemicelluloses, lignin, and rice-husk silica and evaluates the conversion pathways most relevant to domestic implementation. Collectible dry residues from wheat, barley, maize, and rice are estimated at about 15.1 Mt yr^−1^, while potato and cereal starch streams provide directly processable carbohydrates. Near-term options are thermoplastic starch (TPS), cellulose/nanocellulose reinforcement, lignin-containing functional composites, and hybrid materials using rice-husk-derived silica. PLA and PHA offer higher-value routes but require fermentation, purification, polymerization or recovery, and dedicated end-of-life infrastructure. The review emphasizes molecular limitations—hydrogen-bonded polysaccharide networks, retrogradation, interfacial polarity mismatch, lignocellulosic recalcitrance, filler agglomeration, and environment-dependent biodegradation—and links them to processing and performance tests. A staged strategy is proposed: compound and reinforce existing starch streams first, build regional fractionation and standardized intermediate platforms next, and develop PLA/PHA fermentation only after low-cost carbon streams, product markets, and life-cycle advantages are demonstrated.

## 1. Introduction

The transition from petroleum-derived short-life plastics to renewable and biodegradable materials is driven by plastic-waste pressure, fossil-carbon dependence, and the need for regionally resilient material supply chains. Global bio-based plastics still account for a small share of total plastics output, but production capacity is projected to expand substantially toward 2030, with poly(lactic acid) (PLA) and polyhydroxyalkanoates (PHA) among the most visible biodegradable platforms [1]. For Kazakhstan, however, the relevant question is not whether such polymers exist globally, but whether domestic agricultural resources can be connected to technically defensible conversion routes, polymer-grade intermediates, and end-of-life systems.

**Figure 1:**
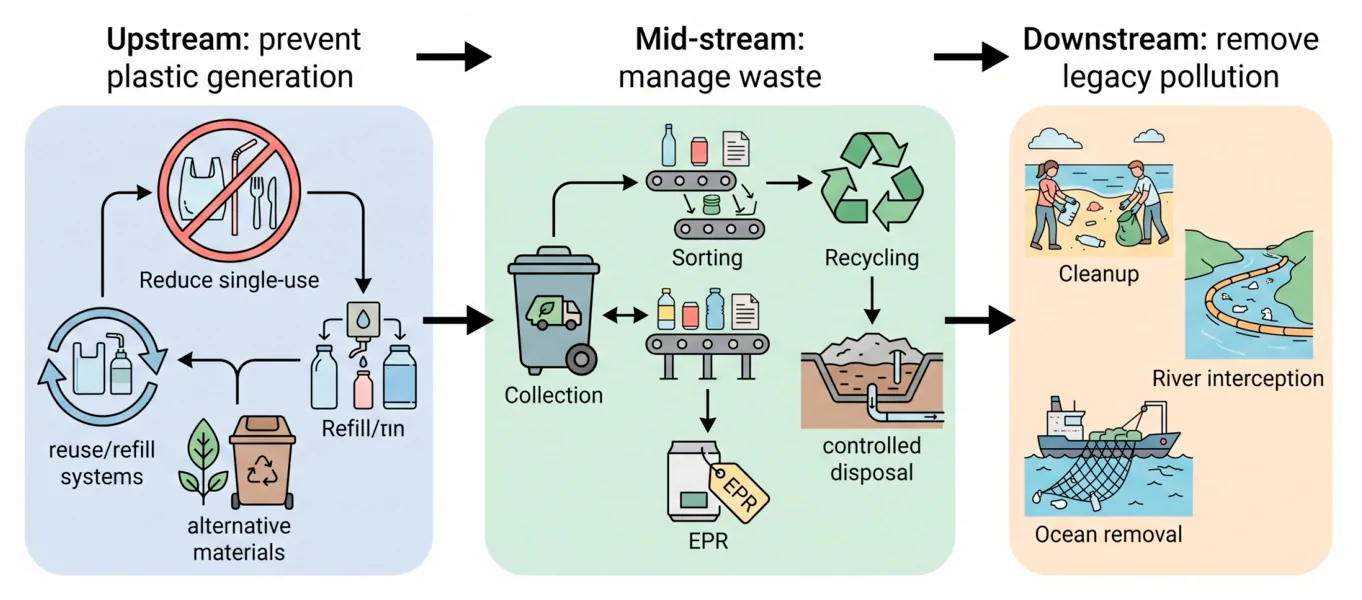
Multi-level strategies for plastic-pollution management. Renewable and biodegradable materials act mainly at the upstream stage, while collection, recycling, extended producer responsibility and removal of legacy pollution remain necessary in parallel. Created in BioRender by Yegissinova, A. (2026). https://BioRender.com/pnco5pb.

The domestic context makes this question practical rather than purely academic. Kazakhstan continues to report limited recovery of several plastic-waste streams, while policy discussions on single-use products have repeatedly exposed a shortage of locally produced alternatives [2,3]. Renewable-material substitution should therefore be treated as one element of a broader hierarchy: prevention and reuse remain preferable where feasible, while recyclable or biodegradable materials are most defensible for short-lived products that are difficult to collect cleanly, such as selected food-contact packaging and agricultural films. Figure 1 places material substitution within this upstream–midstream–downstream framework.

A useful national strategy must distinguish two biomass classes with fundamentally different chemistry. Starch-bearing crops contain semicrystalline granules dominated by amylose and amylopectin and can be converted directly to thermoplastic starch (TPS) or hydrolysed to glucose for fermentation. Lignocellulosic residues are hierarchical composites in which crystalline cellulose microfibrils are embedded in hemicellulose and lignin. Their valorization requires fractionation, because lignin shielding, carbohydrate-lignin associations, and cellulose crystallinity restrict solvent and enzyme access. The resulting process intensity controls chemical consumption, water demand, inhibitor formation, and the economics of downstream polymers.

Unlike broad reviews that catalogue biodegradable polymer families, this work establishes a feedstock-to-polymer framework specifically for Kazakhstan. It links regional biomass availability and composition to pretreatment requirements, molecular-level interfacial problems, conversion routes, testing standards, and staged implementation priorities. The central argument is that the country’s comparative advantage lies not in maximizing one polymer yield from one crop, but in converting co-located starch, cellulose, hemicellulose, lignin, and mineral fractions into a portfolio of polymer-grade intermediates and materials.

The specific contribution of this review is fourfold: it separates Kazakhstan-specific resource and processing evidence from general compositional data; it connects feedstock chemistry to polymer-level limitations such as retrogradation, interfacial polarity mismatch, filler aggregation, and degradation environment; it evaluates existing industrial assets and the missing scale-up links rather than treating laboratory feasibility as industrial readiness; and it converts these observations into stage-gated implementation criteria for Kazakhstan. The novelty therefore lies in the integrated Kazakhstan-specific decision framework rather than in re-cataloguing polymer families already covered by global reviews.

### Scope and Literature Selection

This article is a critical narrative review rather than a systematic review. Evidence was prioritized from peer-reviewed polymer, biomass-conversion and materials-science literature; Kazakhstan-specific agricultural and processing data were taken preferentially from official statistics, peer-reviewed national studies, and clearly identified industry or policy sources where no equivalent primary statistical source was available. Older studies were retained only when they provide distinctive feedstock-specific fractionation or characterization data that remain directly relevant to the conversion chemistry discussed here.

A targeted literature search was conducted in the Scopus database to update the evidence base of this review, covering publications from 2015 to 2026. Broad and topic-specific keyword combinations addressed biodegradable and bio-based polymers, thermoplastic starch, lignocellulosic biomass, cellulose, nanocellulose, lignin, surface modification and compatibilization, rice-husk-derived silica, lactic acid, polyhydroxyalkanoates (PHA), agricultural residues, and Kazakhstan-specific feedstocks. The broad search retrieved 85,397 records, whereas focused searches identified 204 records on lignocellulosic biodegradable polymer systems, 58 on nanocellulose surface modification and compatibility, 22 on rice-husk silica/polymer composites, and 205 on lignocellulosic or agricultural-residue routes to lactic acid or PHA. A Kazakhstan-specific starch/TPS/biopolymer search returned only two records. Because the search sets overlap, the record counts were not summed. The distribution is summarized in Figure S1 (Supplementary Materials). Five recent peer-reviewed studies identified through the targeted update were incorporated to address the principal thematic gaps; additional regionally relevant studies were considered where they directly informed Kazakhstan-specific materials and processing examples.

For reproducibility, the Scopus update was organized around TITLE-ABS-KEY Boolean concept blocks corresponding to the questions addressed in the review: (1) (biodegradable OR bio-based OR bioplastic*) AND (polymer* OR composite*); (2) (lignocellulos* OR cellulose OR nanocellulose OR lignin) AND (biodegradable polymer* OR biocomposite*); (3) nanocellulose AND (surface modification OR compatibilization OR interfac*); (4) “rice husk” AND silica AND (polymer* OR composite*); (5) (lignocellulos* OR “agricultural residue*”) AND (“lactic acid” OR PHA OR polyhydroxyalkanoate*); and (6) Kazakhstan AND (starch OR “thermoplastic starch” OR biopolymer* OR lignocellulos*). Searches were restricted to the 2015–2026 period. For the narrative evidence set, priority was given to peer-reviewed articles and reviews that directly informed feedstock composition, pretreatment/fractionation, polymer conversion, interfacial engineering, biodegradation/end-of-life, or Kazakhstan-specific resource and processing evidence. Focused searches were chosen to close the thematic gaps identified during drafting, rather than to reduce the broad search by a single automated filter.

The 85,397-record result was used only as a scoping indicator of the size of the broader field and was not treated as a PRISMA-style screening pool. Focused result sets were screened for direct relevance to the predefined themes, with overlapping records considered only once when selecting evidence. The statement that five recent studies were incorporated refers to papers newly added during the final targeted update to close specific gaps; it does not mean that only five papers from the broad search underlie the review. Figure S1 is therefore intended to document the update strategy and relative size of the focused search sets, not a complete systematic-review flow diagram.

## 2. Resource Chemistry and Availability in Kazakhstan

**Figure 2:**
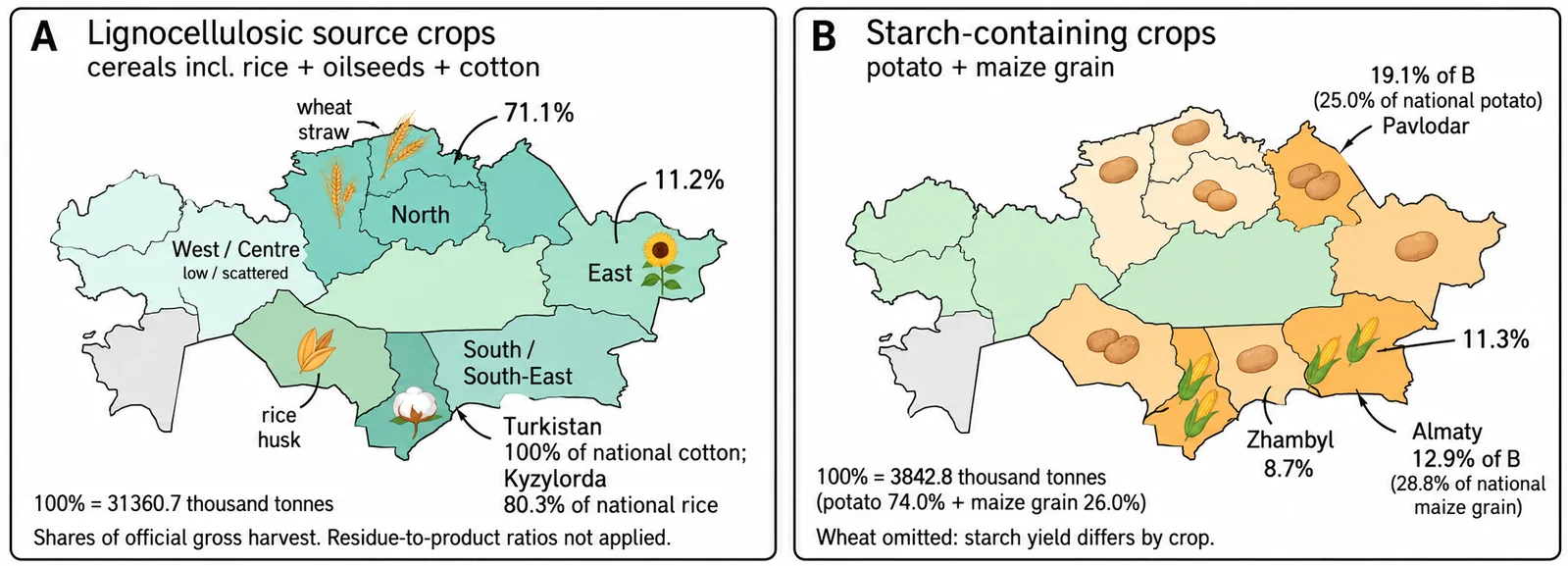
Schematic regional concentration of priority lignocellulosic and starch-containing crops in Kazakhstan. Percentages refer to the crop-group totals used in the original figure and are based on official gross-harvest data [4]. Created in BioRender by Yegissinova, A. (2026). https://BioRender.com/v8eatgi.

Kazakhstan’s agricultural geography creates a useful spatial separation of feedstocks. Northern and northeastern regions dominate cereals; Turkestan concentrates cotton; Kyzylorda dominates rice; and potato and maize are distributed across several northern, southeastern and eastern regions [4,5,6,7,8]. For process design this matters because low-density residues are transport-limited: collection radius, moisture, storage stability and seasonal availability can be as important as nominal cellulose content. Figure 2 summarizes the regional pattern using official gross-harvest data [4].

### 2.1. Cereal Straw and Stover: The Largest Collectible Lignocellulosic Stream

The strongest quantitative case is cereal residue. Using crop production, residue-to-grain ratios, dry-matter factors and availability factors that exclude biomass already committed to other uses, Askarova et al. estimated 15.10 Mt yr^−1^ of collectible dry residue from wheat, barley, maize and rice [9]. Wheat contributes 9.56 Mt yr^−1^, or more than 63% of this total, followed by barley and maize at approximately 2.5 Mt yr^−1^ each. These values are more informative for polymer planning than theoretical residue generation because a fraction of straw must remain for soil organic matter, erosion control, feed and other established uses.

Importantly, the 15.10 Mt yr^−1^ value is a resource-screening estimate of collectible dry residue, not the mass that could be delivered as polymer-grade feedstock. Practical availability must be reduced successively by collection efficiency, storage and dry-matter losses, contamination control, fractionation yield, and purification/recovery yield. Conceptually, the polymer-grade stream can be represented as CB × ηcollection × ηstorage × ηfractionation × ηpurification, where each efficiency term is less than unity and is feedstock- and process-specific. Because Kazakhstan-specific values for all of these efficiencies are not yet available, this review does not convert 15.10 Mt yr^−1^ into a claimed polymer-production tonnage.

The 15.10 Mt yr^−1^ estimate is taken from Askarova et al. [9], who combined crop production with residue-to-grain ratios, dry-matter factors, and availability factors intended to exclude biomass committed to competing uses. The present review adopts that published screening estimate but explicitly treats logistics, storage, fractionation, purification, and polymer-grade recovery as additional downstream constraints.

Chemically, cereal residues provide three value pools. Cellulose is a high-molecular-weight beta-(1→4)-D-glucan that can be converted into regenerated cellulose, microcrystalline cellulose (MCC), cellulose nanofibres (CNF) or cellulose nanocrystals (CNC). Hemicelluloses provide C5/C6 sugar streams, whereas lignin provides an aromatic macromolecular fraction with potential as a UV-active, antioxidant or char-forming component. The complication is recalcitrance: lignin and hemicellulose physically shield cellulose, while the hydrogen-bonded crystalline regions of cellulose resist swelling and hydrolysis [10,11,12].

Wheat is strategically important because grain starch and straw occur in the same value chain. Kazakhstan is a major wheat producer [5], and expansion of deep-grain processing creates an opportunity to couple grain-derived starch or glucose with straw-derived cellulose and lignin [13]. Such integration distributes pretreatment cost across several products and reduces dependence on a single low-value output. Barley and corn residues are smaller in national tonnage but chemically comparable; recent reviews report broad cellulose ranges of roughly 28–46% for barley straw and about 35–50% for corn stalk/stover, emphasizing the variability created by cultivar, anatomy and analytical method [10,14]. The estimated quantities of collectable dry crop-residue biomass in Kazakhstan, differentiated by crop type, are presented in Table 1.

**Table 1:** Calculated collectible dry crop-residue biomass (CB) in Kazakhstan by crop. Cp = grain production; CB = collectible dry biomass. Data from Askarova et al. [9].

Crop	Cp (Mt yr^−1^)	Share of Cp (%)	CB (Mt yr^−1^)	Share of CB (%)
Wheat	12.50	53.40	9.56	63.31
Barley	3.20	13.67	2.55	16.89
Maize	7.20	30.75	2.52	16.69
Rice	0.51	2.17	0.47	3.11
Total	23.41	100.00	15.10	100.00

### 2.2. Cotton Stalk, Rice Husk, and Flax Straw: Regionally Concentrated Residues

Cotton stalk is one of the most chemically favourable lignocellulosic residues in southern Kazakhstan. Cotton cultivation is concentrated in Turkestan, where current policy emphasizes irrigation modernization and integrated processing [6,7]. Representative compositional data report approximately 53.1% cellulose, 14.9% hemicellulose and 19.7% lignin [15], supporting MCC or fibrous reinforcement after pulping, alkali extraction and controlled hydrolysis [16]. Because stalk is generated in the field, its availability is not limited by the utilization rate of cotton gins; the dominant constraints are collection, drying, transport and maintaining enough crop residue on the soil.

Rice husk presents a different chemical problem. Its organic fraction is lignocellulosic, but the ash fraction is unusually high and silica-rich. Kazakhstan-specific studies have demonstrated the production of high-purity amorphous SiO_2_ from local rice husk, including process routes designed to reduce acid use [17,18], and industrial interest is evidenced by a rice-husk-derived silicon-carbon materials line reported in 2024 [19]. This creates a dual-product opportunity: silica can be recovered as a mineral co-product, while the remaining organic fraction can be directed toward cellulose, nanocellulose or carbon-containing composite routes.

Flax straw is smaller in volume but valuable scientifically because Kazakhstan-specific composition has been reported. A Kazakhstan-grown flax straw sample contained approximately 27.4% cellulose, 24.8% hemicellulose and 21.2% lignin [20], whereas fibre-processing studies show that mechanical and chemical history can sharply lower the residual lignin content of the carded fraction [21]. This contrast illustrates a general scale-up problem: process design cannot rely on one textbook composition; cultivar, harvest date, retting, storage and analytical method all alter the apparent fraction distribution.

To avoid conflating national evidence with transferable literature values, the feedstock discussion uses two evidence levels. “Kazakhstan-specific” denotes measurements, production data, or processing demonstrations obtained from Kazakhstan feedstocks or national sources. “General literature” denotes compositional ranges or process behaviour reported for the same crop or material elsewhere and used only as a provisional engineering range. Where local compositional measurements are absent, the general range is not treated as a measured Kazakhstan value and should be verified before process design or scale-up (Table 2).

**Table 2:** Chemical composition of representative lignocellulosic feedstocks relevant to Kazakhstan. Ranges are intentionally broad because composition depends on cultivar, tissue fraction, harvest history and analytical method. * Remainder includes extractives, ash and unresolved fractions where the source did not report them separately.

Feedstock	Cellulose (wt%)	Hemicellulose (wt%)	Lignin (wt%)	Ash/Other	Evidence Basis
Wheat straw	~33–40	~20–30	~12–20	~5–10	General literature range; [10,11]
Barley straw	28–46	17–38	6–19	variable	General literature range; [10]
Corn stalk/stover	35–50	20–35	10–25	variable	General literature range; [10,14]
Cotton stalk	53.1	14.9	19.7	~12.3 *	General literature value; not a Kazakhstan-specific composition measurement; [15]
Rice husk	33–50	15–33	20–30	17–25; ash silica-rich	General composition range + Kazakhstan-specific silica evidence; [10,17,18]
Rice straw	20–57	15–33	6–26	variable	General literature range; [10]
Flax straw (Kazakhstan)	27.4	24.8	21.2	~26.6 *	Kazakhstan-specific feedstock measurement; [20]
Flax fibre/card sliver	—	—	~2.2	—	Kazakhstan-specific processed material; [21]

### 2.3. Starch-Containing Crops and Deep-Grain Processing

Starch offers the shortest chemical route from crop to thermoplastic material. Native granules are semicrystalline assemblies of amylose and amylopectin built from alpha-D-glucose. Their molecular architecture controls gelatinization, melt viscosity, retrogradation, film strength and water uptake. Kazakhstan’s resource base is diversified: wheat and maize support industrial starch production; potato provides a tuber-based stream; and rice contributes a smaller but regionally concentrated source with distinct starch chemistry [22,23,24,25].

Potato is attractive because its processing produces concentrated side streams. Tubers contain a substantial starch fraction [26,27], and potato peel, pulp, washing streams, juice and off-spec tubers can be fractionated into starch, protein, phenolics and fibrous solids. Published studies demonstrate edible/biodegradable films from potato-peel starch, bacterial cellulose production using potato juice, starch foams from potato-processing by-products, and integrated recovery of starch and bioactive compounds [28,29,30,31,32]. These pathways reduce competition with direct food use and are compatible with collection at processing facilities.

The cereal-processing sector is developing in parallel. Reported 2025 output includes approximately 29,000 t of corn starch and 28,000 t of wheat starch, together with gluten, molasses and bioethanol, while major additional deep-grain-processing capacity is planned [13]. From a polymer perspective, the strategic value of this infrastructure is greater than the starch tonnage alone: wet milling, saccharification, syrup handling, fermentation utilities and purification systems are platform assets that could later serve lactic-acid or PHA production. Wheat bran and corn bran add further valorization routes through fibre, arabinoxylan and bioactive fractions [33,34]. Rice by-products likewise provide starch-rich dust, bran and husk streams that can enter film, filler or biorefinery routes [35,36]. The key indicators of starch and starch-crop production in Kazakhstan, together with their potential applications in polymer production, are summarized in Table 3.

**Table 3:** Starch- and starch-crop-relevant indicators and polymer opportunities in Kazakhstan.

Item	Value	Year/Status	Polymer Relevance	Ref.
Potato gross harvest	~2.9 Mt	2024	Starch + concentrated processing residues	[26]
Potato tuber starch	~14–22% fresh mass	reported range	TPS, glucose, films, foams	[27]
Corn starch production	~29,000 t	2025	TPS/fermentable glucose	[13]
Wheat starch production	~28,000 t	2025	TPS/fermentable glucose	[13]
Gluten production	~13,000 t	2025	Potential protein blend/film co-product	[13]
Molasses production	~82,000 t	2025	Potential fermentation carbon stream	[13]
Bioethanol production	~21,000 t	2025	Evidence of fermentation infrastructure	[13]
Planned deep-grain processing	5.8 Mt yr^−1^	by 2028	Platform utilities for starch, sugars and biochemicals	[13]
Planned potato-starch plant (Pavlodar/Aksu)	32,500 t starch yr^−1^; protein 14,500 t; pulp 25,000 t	planned	Dedicated starch and co-product stream	[37]
Rice by-products	regionally concentrated	current	Starch-rich dust + husk silica + fibres	[35,36]
Existing deep-grain-processing facilities	3 operating facilities	2025	Industrial starch/gluten/molasses/bioethanol platform relevant to polymer feedstocks	[13]
Rice-husk Si–C nanocomposite line	1500 t yr^−1^ first-stage target	launched 2024	Commercial-scale residue valorization; filler/material-processing precedent	[19]

### 2.4. Collection, Densification, and Feedstock Logistics

A polymer strategy must account for bulk density as well as chemistry. Loose straw and stalks occupy large transport volume, so long haul distances can erase the economic value of inexpensive feedstock. Local baling, chopping, briquetting or pelleting can increase bulk density and stabilize handling, although densification adds energy consumption and may change moisture behaviour. Modern reviews of biomass densification emphasize the trade-off between transport efficiency, mechanical durability, water sorption and densification energy [38].

For Kazakhstan, decentralized pre-processing is therefore more realistic than transporting untreated residues across long distances. Northern cereal regions could use satellite collection and densification points; cotton stalk should be pre-processed within the Turkestan cluster; and rice-husk processing should remain close to Kyzylorda milling and silica-recovery infrastructure. The logistical objective is not merely to minimize distance, but to deliver a reproducible feedstock specification—particle size, moisture, ash and contamination—compatible with the next chemical step.

## 3. Conversion Chemistry and Polymer Platforms

**Figure 3:**
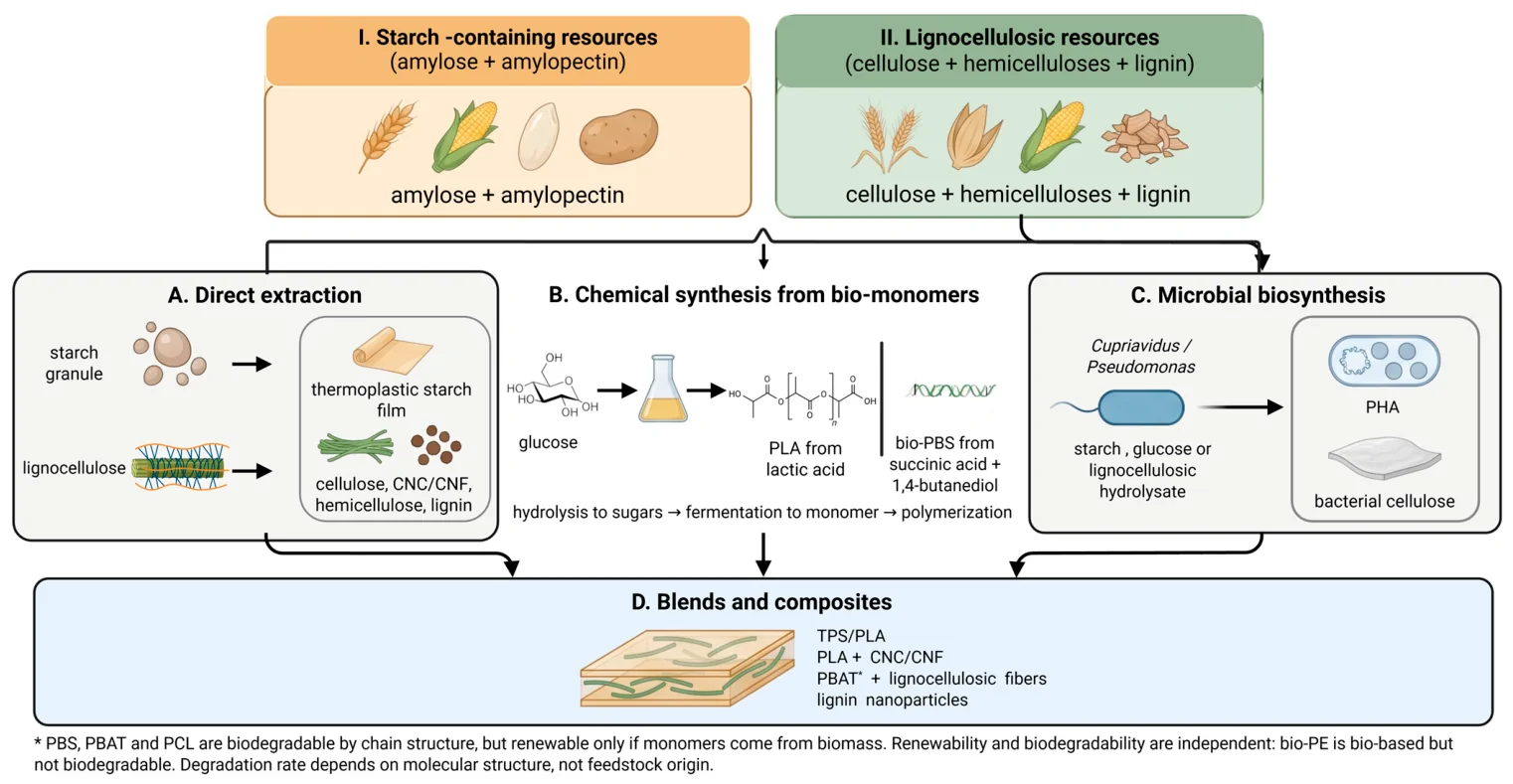
Classification of biodegradable polymer routes obtainable from starch-containing and lignocellulosic feedstocks: direct extraction, chemical synthesis from bio-derived monomers, microbial biosynthesis, and blends/composites. Created in BioRender by Yegissinova, A. (2026). https://BioRender.com/yotq8lr.

The chemistry of the feedstock defines four principal material pathways: direct plasticization of starch; direct recovery and modification of cellulose, nanocellulose and lignin; fermentation of carbohydrate hydrolysates to lactic acid followed by PLA synthesis; and microbial accumulation of PHA. These routes differ in the number of unit operations, sensitivity to impurities, water and solvent demand, and required polymer-processing expertise. Figure 3 summarizes the feedstock-to-material logic and reinforces a critical terminology point: bio-based and biodegradable are not synonymous.

### 3.1. Pretreatment and Fractionation of Lignocellulose

Lignocellulosic pretreatment is an exercise in selective supramolecular disruption. Cellulose contains crystalline domains stabilized by extensive intra- and intermolecular hydrogen bonding; hemicellulose is more amorphous and chemically heterogeneous; lignin is an aromatic macromolecular network that fills the spaces around carbohydrate fibrils and participates in lignin–carbohydrate associations [11,12,39]. An effective pretreatment increases accessible surface area and disrupts lignin shielding without unnecessarily degrading cellulose, condensing lignin or destroying fermentable sugars.

Physical methods such as milling and extrusion mainly reduce particle size. Dilute acids preferentially hydrolyse or solubilize hemicellulose; alkaline treatments promote swelling, ester-bond cleavage and lignin removal; organosolv, deep-eutectic solvents and ionic-liquid systems can produce cleaner fractionation but require solvent recovery. Steam explosion combines thermal, pressure and mechanical effects and can reduce chemical demand, but severe conditions generate furfural, 5-hydroxymethylfurfural, phenolics and organic acids that may inhibit subsequent fermentation [10,11].

Kazakhstan-relevant examples show that the chemistry is tractable at laboratory scale. Wheat straw has been fractionated by multi-level steam-explosion/ethanol processing to recover lignin and hemicellulose while leaving a cellulose-rich fraction suitable for regenerated film [40]. Cotton stalk has been converted to MCC through pulping, alkali extraction and acid hydrolysis [16]. Rice husk has yielded cellulose fibres and CNC after alkaline/bleaching treatment and controlled acid hydrolysis [41,42]. The best pretreatment should therefore be selected by total product value, not by a single cellulose-yield metric.

For polymer applications, fraction yield alone is insufficient to judge pretreatment quality. Cellulose degree of polymerization, crystallinity, fibril dimensions, residual lignin, and surface chemistry influence dissolution/reactivity, fibrillation energy, redispersion, interfacial adhesion, and reinforcement efficiency. Excessive severity can shorten cellulose chains or alter surface functionality, whereas insufficient delignification can impair wetting, colour control, and nanofibrillation. Lignin quality likewise depends on molecular-weight distribution, condensation, retention of beta-O-4 linkages, phenolic/aliphatic hydroxyl content, and residual carbohydrate or ash. Pretreatment studies intended for polymer manufacture should therefore report these quality descriptors together with component yields [11,12,39,43,44,45].

**Figure 4:**
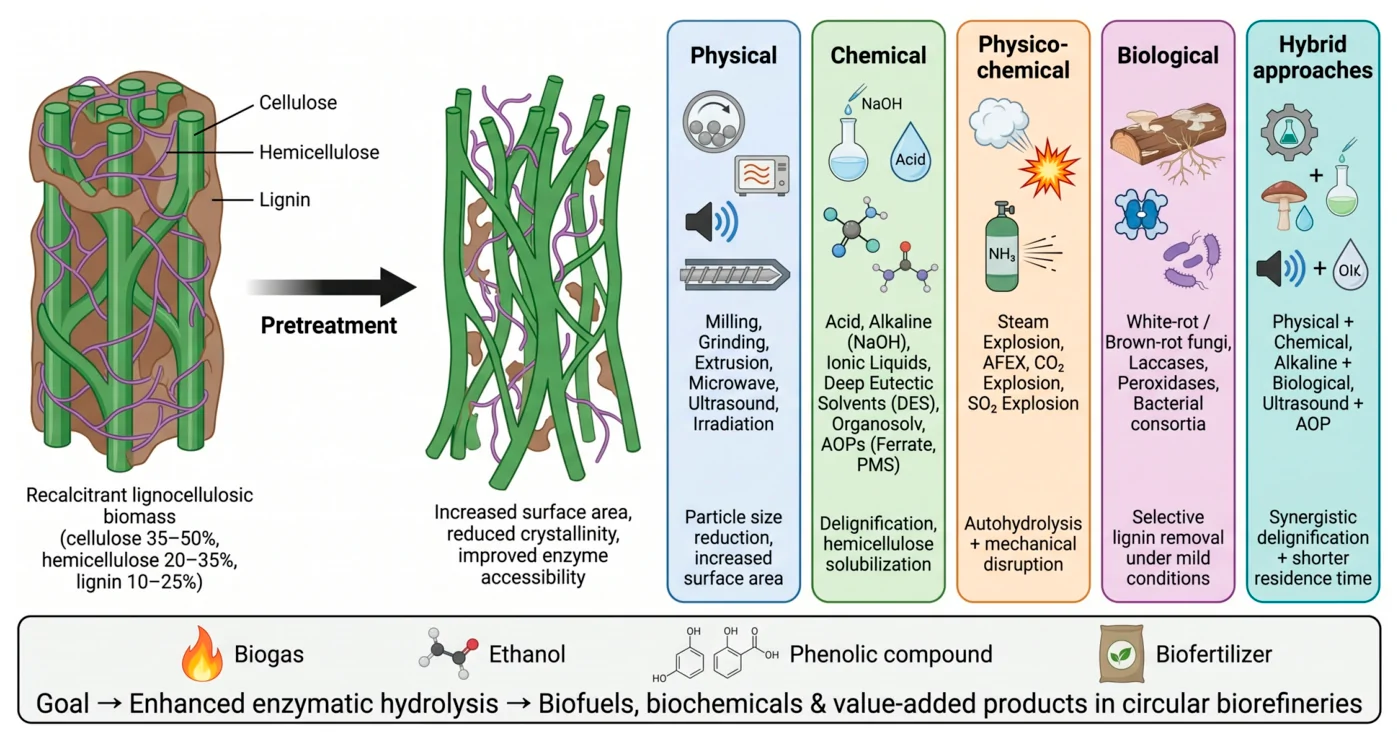
Major pretreatment strategies for lignocellulosic biomass. The preferred route should increase cellulose accessibility while preserving value in hemicellulose, lignin and mineral co-products and minimizing inhibitor formation, solvent loss and water demand [10,11,40]. Created in BioRender by Yegissinova, A. (2026). https://BioRender.com/005wbgj.

From a process-burden perspective, mechanical size reduction uses little process water or reagent but can impose high electricity demand; dilute-acid routes are rapid but require corrosion-resistant equipment, neutralization, washing, and inhibitor control; alkaline routes are effective for delignification but increase chemical consumption, washing demand, and salt-rich wastewater; steam explosion lowers external chemical demand but requires pressurized thermal energy and can generate furans and organic acids; and organosolv, deep-eutectic-solvent, or ionic-liquid routes can produce cleaner cellulose/lignin fractions while shifting burden to solvent cost, recovery, and post-treatment washing. Consequently, pretreatments should be compared using a common feedstock and target product, with chemical consumption, energy, water, inhibitor formation, cellulose preservation, and lignin quality reported together [10,11]. The main pretreatment strategies for lignocellulosic biomass, including physical, chemical, physicochemical, biological, and hybrid approaches, are illustrated in Figure 4. These methods aim to disrupt the lignocellulosic structure, improve cellulose accessibility, and facilitate subsequent enzymatic hydrolysis and conversion into value-added bioproducts.

### 3.2. Cellulose, Nanocellulose, and Lignin as Structural and Functional Components

Cellulose is already a polymer; its conversion challenge is purification, morphology control and interfacial engineering rather than monomer synthesis. The beta-(1→4)-linked glucan backbone gives a stiff chain conformation and extensive hydrogen bonding. Mechanical fibrillation produces high-aspect-ratio CNF, whereas controlled acid hydrolysis preferentially removes less ordered regions to produce CNC. Both can reinforce biodegradable matrices at low loading when dispersion is maintained [12,43].

The same hydroxyl-rich surface that makes cellulose chemically versatile causes its main composite-processing limitation: strong self-association and poor wetting by less polar polyesters. Agglomerates behave as stress concentrators and reduce the effective aspect ratio. Esterification, silanization, grafting, compatibilizers and reactive processing can lower this mismatch, but aggressive substitution may increase cost and can alter degradation behaviour [12,43,44]. Consequently, a Kazakhstan nanocellulose programme should treat surface chemistry, drying/redispersion and compounding as integral parts of the extraction process rather than as downstream afterthoughts.

Lignin should likewise be treated as a functional co-product. Phenylpropanoid units carry phenolic and aliphatic hydroxyl groups and conjugated aromatic structures that can contribute UV absorption, antioxidant activity, rigidity and char formation. Extraction chemistry strongly modifies molecular weight, beta-O-4 content, condensation and residual carbohydrate. Ammonia-hydrothermal fractionation of cotton stalk, for example, yielded lignin rich in beta-O-4 linkages, which is advantageous for later functionalization or depolymerization [45]. In biodegradable composites, controlled lignin loading can therefore serve a stabilizing or barrier-active role rather than merely reduce formulation cost.

### 3.3. Thermoplastic Starch: Molecular Basis of Plasticization and Retrogradation

Native starch is not a conventional thermoplastic because its granules are stabilized by semicrystalline order and a dense network of hydroxyl-group interactions. Under heat and shear in the presence of water and a polyol such as glycerol or sorbitol, water penetrates the granule, crystalline order collapses and amylose/amylopectin chains become sufficiently mobile to form a continuous melt [46,47]. At the molecular level, the plasticizer competes for starch hydroxyl sites, decreases the effective density of starch-starch hydrogen bonding and increases free volume. The result is a lower glass-transition region relevant to processing and improved flexibility.

The same molecular interactions explain the limitations of TPS. Hydroxyl-rich chains readily absorb water, so relative humidity reduces modulus and barrier performance. During storage, mobile amylose and amylopectin segments can reassociate and recrystallize, producing retrogradation, embrittlement and dimensional change [46,47]. More plasticizer increases chain mobility but can increase migration and moisture sensitivity. Higher amylose content can improve film strength but may also change gelatinization and ageing. Kazakhstan-specific starch studies already show source-dependent physicochemical behaviour, confirming that starch origin must be treated as a formulation variable rather than a generic commodity [22]. A recent review of TPS/biodegradable-polyester blends likewise identifies interfacial incompatibility, compatibilization and additive selection as central design variables [48].

For near-term Kazakhstan applications, TPS is most credible in short-life products with moderate structural requirements: produce trays, cushioning foams, nursery pots, selected dry-food packaging and agricultural films. Waste-derived potato starch and cereal-processing side streams are especially attractive because they are co-located with water, energy and drying equipment [28,29,30,31,32]. Recent corn- and potato-starch films demonstrate that source chemistry produces meaningful differences in morphology, thermal behaviour and mechanical response [28].

(C_6_H_10_O_5_)_n_ + nH_2_O → nC_6_H_12_O_6_ (hydrolysis of starch or cellulose to glucose)

### 3.4. Reactive Compatibilization and Hybrid Mineral/Biopolymer Fillers

Blending is the principal route to overcoming the limitations of neat TPS, yet polymer pairs must be treated as multiphase systems. TPS is strongly polar; PLA and many biodegradable polyesters are less hydrophilic. Without compatibilization, interfacial tension produces coarse phase separation, voids and poor stress transfer. In PLA/TPS systems, plasticizer migration can further modify rheology and interfacial behaviour [49].

Reactive compatibilization during extrusion can improve phase morphology by creating covalent or strongly associated links at the interface; anhydride- or epoxy-functional species are common strategies because they can react with starch hydroxyl groups under melt-processing conditions [50]. PBAT/TPS systems likewise show that reactive compatibilizers can refine morphology across strongly dissimilar polarities [51]. In a 2025 TPS/PLA study, combined MAPBAT/EGMA compatibilization increased tensile strain at break from 6% to 73% and reduced the dispersed-domain diameter from 1.35 to 0.35 µm [52].

Lignocellulosic fillers can also be chemically upgraded. Dialdehyde lignocellulose provides aldehyde-rich surfaces that strengthen interactions with TPS and has been shown to improve strength and water resistance relative to unreinforced starch matrices [53]. Nanocellulose surface modification seeks the same outcome—reducing filler–filler association while improving wetting by the matrix. These approaches should be optimized conservatively: excessive functionalization can compromise biodegradability, add reagents and complicate end-of-life claims.

**Figure 5:**
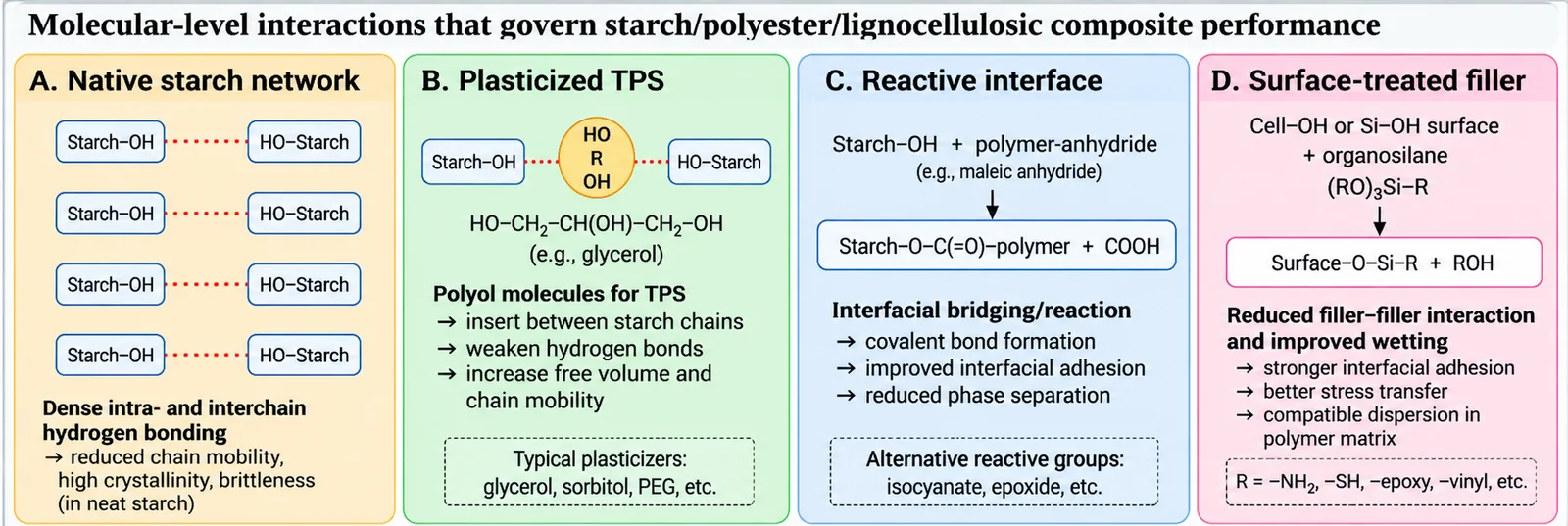
Simplified molecular-level interactions governing starch/polyester/lignocellulosic composite performance: native starch hydrogen bonding, competitive polyol plasticization, reactive interfacial coupling, and organosilane modification of hydroxylated filler surfaces. The reactions are schematic and should be interpreted as mechanistic motifs rather than unique stoichiometric pathways [46,47,49,50,51,53,54,55].

Rice-husk silica requires an analogous interface strategy. High-purity amorphous silica from rice husk carries surface silanol groups that promote particle–particle association. In a polymer matrix, high surface energy and insufficient wetting can produce agglomeration, lowering effective reinforcement. Organosilane treatment or in situ functionalization can modify the silica surface and provide organic groups compatible with the intended polymer; recent work demonstrates one-step functionalization and polymer-grafting potential for rice-husk-derived silica [54], while broader reviews identify particle size, purity and surface chemistry as key determinants of application performance [55]. For Kazakhstan, silica recovery should therefore be coupled to a polymer-specific surface-treatment step rather than treated as a universal filler product. The principal molecular-level interactions governing the performance of starch/polyester/lignocellulosic composites, including native starch hydrogen bonding, polyol plasticization, reactive interfacial coupling, and organosilane surface modification, are schematically illustrated in Figure 5.

### 3.5. PLA: Fermentation Chemistry Followed by Polyester Synthesis

PLA adds a biological conversion step and a chemical polymerization step. Starch or cellulose is hydrolysed to fermentable sugar; microorganisms convert glucose to lactic acid; the acid is purified; and high-molecular-weight PLA is produced most commonly through lactide formation and ring-opening polymerization [56,57,58]. The overall carbohydrate-to-lactic-acid stoichiometry can be represented in simplified form as:

C_6_H_12_O_6_ → 2 CH_3_-CH(OH)-COOH (lactic-acid fermentation)

Polymer performance depends strongly on stereochemistry. L- and D-lactic-acid units control crystallization behaviour, melting characteristics and hydrolysis rate; therefore, fermentation purity and downstream monomer control are scale-up variables rather than analytical details [56,57]. This makes PLA fundamentally more demanding than TPS: a starch plant can produce TPS after extraction, drying, plasticization and extrusion, whereas PLA requires fermentation, purification, oligomerization/lactide production, polymerization and catalyst/process control.

PLA is biodegradable, but rapid mineralization generally requires elevated temperature and humidity such as those provided by industrial composting. Under cooler or drier natural conditions, ester hydrolysis is slow and fragmentation can precede complete biodegradation [59]. Developing PLA without collection and controlled organic-waste treatment would therefore reproduce part of the current waste-management problem in a different polymer. Kazakhstan’s expanding starch/sugar-processing assets nevertheless make PLA a strategically credible long-term platform if high-purity lactic-acid production is established first and polymerization is integrated only after upstream biological conversion is stable.

### 3.6. PHA: Microbial Polymer Biosynthesis from Sugar, Molasses, Lipid, and Hydrolysate Streams

PHA differs from PLA because the polymer is synthesized intracellularly rather than polymerized from an isolated monomer. Microorganisms convert carbon substrates into intracellular polyester granules under controlled nutritional conditions, after which the polymer must be recovered and purified. The PHA family includes short-, medium- and longer-chain hydroxyalkanoate compositions with widely different stiffness, toughness and crystallinity [60]. This compositional flexibility is a major advantage, but strain biology, oxygen transfer, nutrient limitation, fermentation control, cell disruption and polymer recovery all contribute to cost.

For Kazakhstan, substrate economics are decisive. Refined glucose offers reproducibility but may be too valuable for a commodity polymer. Molasses, starch-processing residues, waste lipids and lignocellulosic hydrolysates become more attractive if inhibitory compounds can be controlled [60,61]. PHA has a broader biodegradation envelope than PLA, with documented degradation in soil, compost, freshwater, marine, sludge and anaerobic environments depending on polymer composition and conditions [62,63]. That profile is attractive for difficult-to-collect short-life products, but fermentation and downstream recovery requirements make PHA a medium- to long-term target rather than the first domestic platform.

### 3.7. Comparative Feedstock-to-Polymer Matching

The conversion hierarchy is therefore chemically clear: TPS requires the fewest transformations; cellulose/nanocellulose requires intensive fractionation but no monomer synthesis; PLA requires hydrolysis, fermentation and chemical polymerization; PHA requires fermentation and intracellular polymer recovery; and hybrid composite routes add an interfacial-engineering step. Table 4 translates this hierarchy into Kazakhstan-specific opportunities and constraints.

To avoid implying quantitative precision that is not supported by comparable Kazakhstan-specific TEA/LCA data, Table 4 presents the route comparison qualitatively. For energy demand (E), process-water demand (W), cost/capital burden (C), and yield/recovery penalty (Y), the descriptors low, moderate, and high reflect the number and severity of major unit operations documented for each route: direct melt processing or simple compounding is treated as a lower burden; fractionation, surface treatment, or intensive purification as a moderate burden; and fermentation combined with purification, polymerization, or intracellular polymer recovery as a higher burden. Kazakhstan-specific readiness (K) is classified as high when both domestic feedstock and relevant processing infrastructure are already available, moderate when feedstock and partial processing capability exist but polymer-specific integration remains to be demonstrated, and low when a dedicated production chain and scale-up evidence are absent. These categories are screening descriptors synthesized from the process steps and literature cited for each route and are not substitutes for measured national TEA/LCA results.

## 4. Cross-Cutting Materials Performance, Biodegradation, and Sustainability

### 4.1. Interfacial Compatibility and Moisture

The central materials-science problem across starch- and cellulose-rich systems is polarity mismatch. TPS, cellulose and nanocellulose contain dense hydroxyl functionality and interact strongly with water, whereas PLA and several biodegradable polyesters are less polar. Without compatibilization, multiphase blends display weak stress transfer, interfacial voids, coarse domains and rapid property loss at elevated humidity [43,49,50,51]. This means that dry tensile strength alone is an insufficient development metric.

A practical Kazakhstan programme should condition specimens at controlled relative humidity and report mechanical properties together with water uptake, water-vapour transmission, oxygen permeability, dimensional stability and ageing. For fillers, dispersion should be quantified by microscopy or scattering rather than inferred only from strength. For TPS-containing materials, repeated humidity cycling is especially important because plasticizer redistribution and retrogradation can change properties after the initial test.

### 4.2. Performance Windows and Minimum Testing

Different applications require different property windows. Agricultural films need tear resistance, wet–dry cycling tolerance, UV stability and a predictable soil-degradation period; high optical clarity and extremely low oxygen transmission may be secondary. Food-contact packaging can require migration control, moisture and oxygen barriers, sealability, grease resistance and tighter dimensional stability. Reinforced trays and foams require compressive or flexural performance rather than the same tensile targets as thin film. Table 5 therefore emphasizes a minimum testing framework rather than universal numerical thresholds.

### 4.3. Biodegradation Must Be Matched to the Intended Environment

Terminology is used consistently as follows: bio-based describes the origin of polymer carbon and does not by itself imply biodegradation; biodegradable describes biologically mediated conversion under specified environmental and test conditions; and compostable denotes compliance with defined composting requirements, including biodegradation/disintegration and, where applicable, compost-quality criteria. A material should therefore not be described simply as “biodegradable” or “compostable” without identifying the relevant environment or standard [59,62,63,64,65,66,67].

Disintegration and biodegradation are not interchangeable. Visual fragmentation demonstrates loss of physical integrity, whereas biodegradation requires microbial assimilation and conversion of polymer carbon to carbon dioxide, water and biomass under aerobic conditions or to carbon dioxide, methane and biomass under anaerobic conditions. The rate depends on chemical structure, molar mass, crystallinity, morphology, additives, temperature, moisture and the microbial community [59,62,63,68,69].

For industrial composting, ISO 14855-1 provides a controlled method based on evolved carbon dioxide and disintegration [64]; ASTM D6400 specifies requirements for plastics intended for aerobic municipal or industrial composting facilities [65]; and EN 13432 addresses compostability of packaging through biodegradability, disintegration, process effects and compost quality [66]. Agricultural mulch films require a different framework: EN 17033 specifies requirements and test methods for thermoplastic films intended to biodegrade in soil without adverse environmental effects [67]. These distinctions are important for Kazakhstan because a material designed for industrial composting should not be marketed as if it will disappear rapidly in cold soil or uncontrolled open environments.

For PLA-rich systems, industrial-composting testing is the most defensible first claim unless a different route has been demonstrated experimentally. TPS/cellulose systems can additionally be assessed in soil or compost, whereas PHA may justify broader environmental testing depending on polymer composition and product use [62,63]. End-of-life should therefore be specified before formulation, not added as a label after mechanical testing.

Industrial-composting evidence must not be extrapolated to home composting, agricultural soil, freshwater, marine water, landfill, or uncontrolled ambient litter. These environments differ in temperature, moisture, oxygen supply, microbial activity, and residence time. Soil-oriented claims require soil-specific testing, while aquatic degradation claims require separate environment-specific methods; neither ASTM D6400 nor EN 13432 demonstrates rapid degradation in those settings. Accordingly, end-of-life statements in this review are tied to the intended disposal environment rather than to a generic material label [62,63,64,65,66,67].

At the reaction level, PLA degradation proceeds through ester-bond hydrolysis. Water uptake generates hydroxyl- and carboxyl-terminated chains; the newly formed carboxyl groups can lower the local pH and accelerate further hydrolysis, producing the well-known autocatalytic contribution to bulk degradation. The balance between water diffusion and chain scission depends on specimen thickness, molar mass, crystallinity, temperature relative to Tg, and the mobility of amorphous segments [59,69]. Consequently, a rapid loss of molar mass does not necessarily imply equally rapid mass loss or mineralization. This distinction is important when interpreting composting data for thick moulded articles versus thin films.

PHA degradation is chemically related but biologically distinct. Extracellular or cell-associated depolymerases can adsorb at the polymer surface and cleave ester bonds, after which soluble oligomers and hydroxyalkanoate monomers are assimilated. Surface accessibility, crystallinity and monomer composition therefore control rate; amorphous regions are generally more susceptible than densely packed crystalline domains [62,63]. TPS-containing systems introduce a third mechanism because starch can be enzymatically depolymerized through alpha-glucosidic bond cleavage while the polyester phase follows its own hydrolytic or enzymatic pathway. In blends, disappearance of starch can increase porosity and water access, so apparent disintegration can accelerate even when the polyester fraction remains only partly mineralized [68].

### 4.4. Pretreatment Inhibitors, Water Demand, and Co-Product Recovery

Lignocellulose processing shifts the main challenge from polymer physics to separation chemistry. Harsh acid conditions generate furans and organic acids; alkaline systems produce salt-rich wastewater; organosolv routes require solvent recovery; and most wet routes consume water. This is especially important in Kazakhstan because several promising biomass streams are located in water-stressed regions. Closed-loop washing, counter-current extraction, solvent recovery and high-solids processing should therefore be treated as design requirements rather than later optimization steps.

Co-product recovery can change the process balance. Rice-husk silica can offset the cost of de-ashing [17,18]; lignin can become a saleable functional fraction instead of a low-value waste; and hemicellulose-derived sugars can supply fermentation or platform-chemical routes. A fractionation process that gives a slightly lower cellulose yield but preserves clean lignin, fermentable hemicellulose sugars and recoverable silica may be economically superior to a cellulose-maximizing route that destroys or contaminates the remaining fractions.

### 4.5. Feedstock Variability and Polymer-Grade Specifications

Industrial polymer production requires tighter feedstock specifications than combustion, bedding or bulk feed. Moisture, ash, lignin, extractives, cellulose crystallinity, starch amylose/amylopectin ratio, particle size, microbial contamination and storage history all influence processing and product quality. Kazakhstan-specific data remain sparse for several residues, and the differences already observed in flax demonstrate the risk of applying imported average values to local process design [20,21].

A national feedstock database should therefore use standardized analytical methods and report composition by crop, cultivar, region, harvest year and storage condition. For lignocellulose, structural carbohydrates, acid-insoluble/soluble lignin, ash and extractives should be reported separately; for starch, amylose content, gelatinization profile, moisture, protein/lipid impurities and particle morphology are required. The output of this programme should be polymer-grade feedstock specifications, not only agricultural statistics.

### 4.6. Life-Cycle and Techno-Economic Constraints

Renewable carbon is not automatically equivalent to a lower environmental footprint. PLA life-cycle studies show large variability because results depend on feedstock, electricity mix, system boundary, biogenic-carbon accounting and end-of-life assumptions. A 2024 review and meta-analysis reported a lower median climate impact for cradle-to-gate PLA resin than for several fossil polymers, but cradle-to-grave results were much less clearly advantageous, demonstrating the sensitivity to use and disposal scenarios [70]. A broader 2024 review of bioplastics likewise emphasizes land use, fertilizer inputs, process energy, collection, and incomplete degradation as variables that can offset part of the feedstock benefit [71].

This is particularly relevant in Kazakhstan, where electricity and process heat may carry a substantial fossil-energy burden and irrigated agriculture can be water-intensive. Energy-intensive drying, solvent recovery, repeated washing or lactide purification can therefore dominate the footprint of an otherwise renewable route. For this reason, every pilot programme should pair materials characterization with techno-economic analysis (TEA) and cradle-to-grave life-cycle assessment (LCA). The preferred option should be the route that delivers required performance with the lowest combined cost, water demand, energy demand and end-of-life burden—not necessarily the route with the highest bio-based content.

A robust comparison should use the same functional unit and service requirement for the bio-based and incumbent material. For a mulch film, for example, the functional unit should represent protected area and service duration rather than simply one kilogram of polymer. For packaging, barrier performance and product-loss prevention can be more important than resin mass alone. This functional-performance perspective is essential to avoid misleading comparisons.

For Kazakhstan-specific pilot assessment, a minimum TEA/LCA dataset should include feedstock gate price, collection radius and bulk density, baling/densification energy, storage loss, fractionation and purification yields, reagent consumption and recovery, electricity and thermal-energy demand, process water and wastewater treatment, product purity and yield, co-product credits, capital and operating costs, and the actual end-of-life scenario. Reporting these variables on the same functional basis is more informative than importing isolated cost or energy numbers from studies with different feedstocks and system boundaries. This common-data approach is especially important when comparing a low-complexity TPS route with fractionation-intensive cellulose systems or fermentation/recovery-intensive PLA and PHA routes [70,71].

### 4.7. Current Industrial Baseline and Translation Gap in Kazakhstan

Kazakhstan already has an industrial upstream base relevant to biopolymer development, although it is not yet a dedicated biodegradable polymer value chain. In 2025, three operating deep-grain-processing facilities produced approximately 29,000 t of corn starch, 28,000 t of wheat starch, 13,000 t of gluten, 82,000 t of molasses, and 21,000 t of bioethanol, while additional deep-processing capacity totaling 5.8 Mt yr^−1^ was reported as planned by 2028 [13]. These assets are directly relevant because they provide starch/sugar streams and industrial experience in wet milling, separation, fermentation utilities, and purification.

Industrial translation is also visible outside grain processing. A rice-husk-derived silicon-carbon nanocomposite production line was launched with a first-stage target of 1500 t yr^−1^, demonstrating commercial-scale residue valorization and a potential filler/material platform [19]. A dedicated potato-starch project has also been reported as planned, with starch, protein, and pulp co-product streams [37]. These examples support the feasibility of feedstock processing, but they should not be interpreted as evidence that a domestic PLA or PHA manufacturing chain already exists.

The sources reviewed for this article document upstream agricultural processing and selected residue-valorization assets, but not a fully integrated domestic chain covering polymer-grade fractionation, PLA/PHA synthesis or recovery, application-scale compounding, certified end-of-life infrastructure, and stable product off-take. The proposed roadmap therefore separates existing industrial capability from proposed polymer production: near-term recommendations build on documented starch/filler and processing assets, whereas fermentation-based polymer routes remain conditional on pilot-scale product quality, economics, environmental performance, and end-of-life capacity [13,19,37].

## 5. A Staged Implementation Strategy for Kazakhstan

The deployment classes in this section were assigned using six explicit criteria: (i) availability of a documented domestic feedstock or side stream; (ii) number and severity of conversion and recovery operations; (iii) compatibility with existing Kazakhstan processing assets; (iv) need for new fermentation, polymerization, solvent-recovery, or intracellular-polymer-recovery capacity; (v) expected process-control and cost burden; and (vi) availability of a credible end-of-life route for the intended product. “Near term” therefore denotes routes that can be piloted largely with existing starch, filler, and conventional compounding/processing capability, whereas “medium” and “long term” categories require progressively more new fractionation, biological conversion, purification, polymer synthesis/recovery, and end-of-life infrastructure.

These labels are planning classes rather than forecasts. The time windows in Table 6 are subordinate to the stated go/no-go gates: a route should advance only after pilot yield and product specifications, process stability, TEA/LCA, end-of-life compliance, and market/off-take are demonstrated. Conversely, a route may move earlier or later than the indicative window if those conditions are met sooner or remain unresolved.

### 5.1. Near Term: TPS, Reinforcement, and Hybrid Fillers

The lowest-risk entry point is a materials platform that uses infrastructure already present in food and agricultural processing. Native wheat, corn or potato starch can be plasticized and extruded into TPS, while wheat/barley straw, cotton stalk, flax fractions or rice-husk-derived cellulose can provide fibrous or nanoscale reinforcement. This route avoids the need to build a dedicated fermentation industry at the outset. The technical programme should focus on formulation windows, compatibilizers, water resistance, conditioned mechanical properties and processability on conventional film, sheet and moulding equipment [22,28,43,46,47,49,50,51,53].

The first products should have short service lives and manageable performance requirements: agricultural mulch films, nursery pots, protective packaging, loose-fill/foamed packaging, produce trays and selected dry-food packaging. Lignin-rich fractions can be screened as UV-active or antioxidant additives, while rice-husk silica can be developed as a surface-treated reinforcing or barrier filler [54,55]. The most attractive products are those that consume multiple fractions of the same residue stream and avoid creating a new low-value waste fraction. Regional work provides useful proof-of-concept examples for this near-term materials pathway. Egamberdiev and co-workers have reported cellulose nanocrystals recovered from secondary paper waste and composite papers based on plant-derived cellulose, demonstrating regional capability in cellulose recovery, nanoscale reinforcement, and natural-polymer composite processing [72,73]. Kazakhstan-based studies involving Nurlybayeva have also examined cellulose–chitosan biocomposites and pectin-based biodegradable packaging from fruit-peel waste, directly supporting the feasibility of renewable polysaccharide materials for packaging-oriented applications [74,75]. In parallel, a recent solvent-free P(MMA-AA) study involving Nurlybayeva and Kussainova illustrates regional experience with lower-solvent polymer processing and structure–property evaluation; because the acrylic copolymer is not biodegradable or biomass-derived, it is cited here only as a processing precedent rather than as a target material in the present roadmap [76].

### 5.2. Medium Term: Regional Fractionation and Standardized Intermediate Platforms

Once collection logistics and polymer compounding are established, the next step is integrated regional fractionation. Northern grain regions are suited to cereal-straw fractionation; Turkestan to cotton-stalk processing; Kyzylorda to combined rice-husk silica and cellulose recovery; and potato/starch residues to processing-centred biorefineries. The principal outputs should be standardized intermediate streams—cellulose pulp, CNC/CNF, lignin, glucose syrup, C5-rich hydrolysate and mineral silica—each with specifications suitable for several downstream customers.

This platform logic reduces technology lock-in. Glucose, cellulose pulp, lignin and silica retain independent markets even if demand for one polymer changes. It also creates a practical quality-control pathway: intermediate products can be certified and traded before Kazakhstan commits to an integrated PLA or PHA plant.

### 5.3. Long Term: PLA and PHA as Fermentation-Based Targets

Dedicated PLA and PHA production becomes rational when three conditions are met: a stable low-cost carbon stream, fermentation/purification expertise, and a credible domestic or export market. Planned deep-grain-processing expansion can contribute to the first condition [13], but polymer-grade biological conversion still requires dedicated development. PLA is likely to be easier to stage because lactic-acid fermentation and chemical polymerization can be separated into distinct industrial steps. PHA offers broader environmental degradability but requires tighter integration of strain selection, fermentation control and polymer recovery [56,57,59,60,62,63].

For both routes, LCA and TEA should precede commercial scale-up. A residue-fed process that sells lignin and silica co-products may outperform a simpler food-grade-starch pathway, while a high-purity polymer route powered by carbon-intensive heat may lose much of the expected climate advantage [70,71]. Kazakhstan’s comparative advantage will therefore depend on integration and energy strategy rather than feedstock abundance alone.

### 5.4. Market and Policy Alignment

Policy should distinguish renewable carbon content from verified end-of-life performance. A starch-filled conventional polyolefin is not automatically biodegradable, and a biodegradable polyester is not necessarily bio-based. Claims should be tied to recognized test conditions and standards. At the same time, incentives should favour secondary agricultural streams where they can provide equivalent function, because residue valorization creates an additional environmental benefit beyond material substitution.

Predictable demand is equally important. Procurement standards for public institutions, agricultural-film pilots, packaging specifications, extended-producer-responsibility incentives and transparent compostability/biodegradability labelling can create an initial market without requiring abrupt economy-wide substitution [2,3]. The aim should be a performance-based market signal: materials that meet service and end-of-life requirements should be rewarded regardless of whether they originate from one particular polymer family.

### 5.5. Future Research Directions and Priority Roadmap

Future research should move beyond feedstock inventories and isolated laboratory films toward integrated feedstock-to-material validation under Kazakhstan-specific conditions. The first priority is standardization: cellulose, hemicellulose, lignin, ash, extractives, starch content, amylose/amylopectin ratio, moisture, and seasonal variability should be measured by harmonized methods so that polymer-grade acceptance windows can be defined for regional residues. In parallel, pretreatment research should compare not only cellulose yield but also water and solvent recovery, inhibitor formation, lignin quality, hemicellulose-sugar recovery, and silica recovery from rice husk. Interfacial engineering should be treated as a molecular-design problem, linking compatibilization or surface functionalization to dispersion, humidity-conditioned mechanics, barrier properties, and biodegradation [10,11,12,43,49,50,51,53,54,55].

A second priority is translation from laboratory-scale proof of concept to pilot processing and application-specific validation. Extrusion, blown-film processing, thermoforming, foaming, and compounding should be assessed at realistic throughput, followed by testing under the intended service environment and recognized end-of-life standards. Fermentation-based PLA and PHA routes should advance only after low-cost hydrolysates, inhibitor control, purification, and polymer recovery are demonstrated at pilot scale. Each scale-up step should be screened by Kazakhstan-specific LCA and TEA using realistic electricity, heat, water, irrigation, transport, residue-collection, and co-product assumptions [56,57,59,60,62,63,64,65,66,67,68,69,70,71]. Table 6 provides the time-phased implementation context; the priorities below define the corresponding research agenda.

1. Establish a Kazakhstan feedstock atlas using harmonized protocols for structural carbohydrates, lignin, ash, extractives, starch, amylose/amylopectin ratio, moisture, and seasonal variability.

2. Identify low-water, recyclable pretreatment sequences that maximize total product value, including lignin, hemicellulose sugars, and rice-husk silica rather than cellulose yield alone.

3. Determine which Kazakhstan starch sources provide the best TPS processing/retrogradation balance and which compatibilization strategies improve water resistance and interfacial adhesion without undermining biodegradability.

4. Develop polymer-specific surface treatments for CNF/CNC and rice-husk silica and quantify their effects on dispersion, barrier performance, mechanical durability, and end-of-life behaviour.

5. Test hydrolysates from straw, cotton stalk, molasses, and potato-processing residues for lactic-acid and PHA fermentation, including inhibitor tolerance, purification, and recovery cost.

6. Define product-specific end-of-life pathways and validate claims using ISO 14855-1, ASTM D6400, EN 13432, EN 17033, or other environment-appropriate standards rather than generic ‘biodegradable’ labels.

7. Couple pilot-scale materials data with Kazakhstan-specific LCA/TEA before selecting a commercial route, using these analyses as stage-gates rather than post hoc justification.

Together, these directions shift the field from proof-of-concept bioplastics toward reproducible polymer engineering in which local feedstock chemistry, processing, material performance, standardized biodegradation, and scale-up metrics are reported within one comparable framework.

## 6. Conclusions

Kazakhstan does not lack renewable carbon; it lacks an integrated route from agricultural feedstock to polymer-grade intermediate and finished material. The collectible cereal-residue base alone is about 15.1 Mt yr^−1^, and this is complemented by regionally concentrated cotton stalk, rice husk, flax straw, potato, and expanding wheat/corn processing. The practical value of these resources depends on their molecular differences: starch is a direct thermoplastic precursor after plasticization but remains moisture-sensitive and prone to retrogradation; cellulose and nanocellulose are strong reinforcements but require fractionation and interface control; lignin can become a functional UV/antioxidant phase; rice-husk silica is a valuable mineral co-product if particle size and surface chemistry are controlled; and PLA/PHA require fermentation, purification and end-of-life infrastructure beyond what is currently established domestically.

The most credible development sequence is therefore staged. Near-term work should prioritize TPS, reinforced polysaccharide materials and hybrid lignocellulose/silica composites using existing starch and agricultural-residue streams. Medium-term investment should build regional fractionation and standardized intermediate-product platforms. Longer-term projects should integrate lactic-acid/PLA and PHA production only after low-cost carbon streams, market demand and Kazakhstan-specific LCA/TEA advantages are demonstrated. The decisive metric is not maximum polymer yield from one crop, but maximum functional and economic value recovered from the full biomass through coordinated carbon, fibre, lignin and mineral utilization.

This approach also changes the research objective. Instead of asking whether Kazakhstan can produce a biodegradable polymer in the laboratory, the more useful question is whether a defined local feedstock can be reproducibly converted, compounded, tested against an application-specific standard, and recovered or biodegraded through an available end-of-life route at acceptable cost and environmental burden. Answering that question requires polymer chemistry, feedstock analytics, process engineering, logistics, standards and life-cycle assessment to be developed as one integrated programme.

## Figures and Tables

**Table 4:** Comparative feedstock-to-polymer matching and qualitative route screening for Kazakhstan. The additional rows distinguish reinforced TPS, biodegradable-polyester/TPS blends and rice-husk-silica composites, because these hybrid systems are plausible transitional products between raw biomass valorization and dedicated fermentation-based polymer manufacture.

Route	Primary Feedstock	Key Process Steps	Principal Kazakhstan Constraint	Indicative Deployment/Process Route	Qualitative Screening **	Refs.
Thermoplastic starch (TPS)	Potato, corn, wheat or rice starch	Gelatinization/plasticization; extrusion or film forming	Moisture sensitivity, retrogradation, moderate strength	Near term; direct starch plasticization and melt/film processing	E: Low; W: Low; C: Low; Y: Low; K: High	[22,46,47]
Reinforced TPS	TPS + cellulose/CNF/CNC/lignocellulose	Compounding; dispersion; optional surface treatment	Filler aggregation; interface control; water uptake	Near term; compounding with optional filler surface treatment	E: Low; W: Low; C: Low; Y: Low; K: Moderate	[43,53]
PLA/TPS or PBAT/TPS blends	Starch + biodegradable polyester	Melt blending + compatibilization	Polarity mismatch; phase separation; plasticizer migration	Near–medium term; melt blending and reactive/physical compatibilization	E: Low; W: Low; C: Low; Y: Low; K: Moderate	[49,50,51]
Cellulose/nanocellulose	Cotton stalk, wheat/barley straw, rice husk, flax	Delignification/fractionation; fibrillation or hydrolysis	Pretreatment cost; drying/redispersion; matrix compatibility	Near–medium term; multistep fractionation followed by fibrillation or hydrolysis	E: High; W: High; C: High; Y: Moderate; K: Low	[16,40,41,42,43,45]
Lignin functional phase	Co-product of fractionation	Alkaline/organosolv/hydrothermal isolation; optional modification	Variable purity, colour and molecular structure	Near–medium term; fractionation/isolation with optional functional modification	E: Moderate; W: High; C: Moderate; Y: Moderate; K: Low	[45,53]
Rice-husk silica composite	Rice husk ash/recovered silica	Silica recovery; particle control; silanization or grafting; compounding	Agglomeration; surface chemistry; co-product integration	Near–medium term; silica recovery, particle control, surface treatment and compounding	E: High; W: Moderate; C: Moderate; Y: Low; K: Moderate	[17,18,54,55]
PLA	Starch hydrolysate or lignocellulosic C6 sugars	Saccharification → lactic acid → lactide → ROP	No dedicated domestic PLA chain; purity and capital intensity	Medium–long term; saccharification, fermentation, lactic-acid purification, lactide formation and polymerization	E: High; W: High; C: High; Y: High; K: Low	[56,57,59]
PHA/PHB	Sugars, molasses, lipids, hydrolysates	Microbial fermentation → accumulation → recovery	Fermentation know-how; recovery cost; feedstock consistency	Long term; microbial fermentation, intracellular accumulation, cell disruption, recovery and purification	E: High; W: High; C: High; Y: High; K: Low	[60,62,63]

** Qualitative screening descriptors: E = relative energy demand; W = relative process-water demand; C = relative cost/capital burden; Y = yield/recovery penalty; K = Kazakhstan-specific readiness. Burden categories are reported as low, moderate, or high according to the process complexity and unit operations documented in the cited literature; readiness is reported as low, moderate, or high according to the presence of domestic feedstock, relevant industrial assets, and polymer-specific scale-up evidence. The comparison is intended for route screening only; pilot-scale TEA/LCA using a common functional unit is required for quantitative ranking.

**Table 5:** Application-specific performance and testing framework for biodegradable polymer systems relevant to Kazakhstan. The table deliberately avoids one universal mechanical threshold because acceptable performance depends on product geometry, conditioning and service environment.

Material/Application	Critical Polymer Property	Minimum Test Set	Key Failure Mode to Watch	Standard/Guidance
TPS films/dry-food packaging	Tensile strength; elongation; water-vapour barrier; ageing	Tensile test; WVP; water uptake; DSC/DMA; humidity conditioning	Plasticizer migration; retrogradation; moisture softening	Application-specific; compostability claim only after validated biodegradation
PLA/TPS or PBAT/TPS film	Interfacial adhesion; tear/elongation; barrier; morphology	Tensile/tear; SEM; DSC; WVP/O_2_ permeability	Coarse phase separation; interfacial voids	Use compostability standards where label is claimed [64,65,66]
CNF/CNC-reinforced biocomposite	Dispersion; modulus; toughness; moisture uptake	Microscopy; tensile/flexural; DMA; WVP; contact angle	Agglomeration; embrittlement; moisture-driven interface loss	No single universal threshold; report conditioned properties
Rice-husk-silica composite	Particle dispersion; interface; barrier; thermal stability	Particle-size/SEM; FTIR; tensile; TGA/DSC; WVP	Silica agglomeration; poor wetting; brittle fracture	Surface treatment must be matched to matrix [54,55]
Industrial-compostable packaging	Disintegration + mineralization + compost quality	Controlled composting + CO_2_ evolution + disintegration	Fragmentation without sufficient mineralization	ISO 14855-1; ASTM D6400; EN 13432 [64,65,66]
Biodegradable mulch film	Service life in soil; tensile retention; ecotoxicity; soil biodegradation	Ageing + soil biodegradation + plant/soil organism effects	Premature loss in service or persistent fragments	EN 17033 [67]
PHA short-life products	Mechanical properties + environment-specific degradation	Tensile/barrier + soil/compost/aquatic tests as relevant	Assuming all PHA grades degrade equally fast	Select test environment according to intended disposal [62,63]

**Table 6:** Proposed staged research-to-implementation roadmap. Time windows are indicative planning horizons, not forecasts; each stage should proceed only when its technical and environmental gate is met. The horizons reflect domestic readiness and required infrastructure; they are not predictions of commercial launch dates.

Stage	Regional Feedstock Focus	Intermediate/Material Target	Critical Technical Gate	Go/No-Go Decision Metric
0–2 years	Existing starch streams; wheat/barley straw; cotton stalk	TPS compounds; fibres; MCC; initial lignin/silica fillers	Reproducible feedstock specification; extrusion window; humidity-conditioned properties	Stable formulation on conventional equipment with defined disposal route
2–5 years	Northern cereal residues; Turkestan cotton; Kyzylorda rice husk	CNF/CNC; purified lignin; functionalized rice-husk silica; glucose/C5 hydrolysates	Fractionation yield + solvent/water recycle + co-product quality	Positive integrated mass balance and pilot TEA/LCA
4–7 years	Deep-grain processing and selected residue hydrolysates	Polymer-grade lactic acid; fermentation-grade carbon streams	Purity, inhibitor control, fermentation productivity and purification cost	Lactic acid competitive in more than one market before polymer integration
5–10 years	Integrated starch/residue biorefineries	PLA and selected PHA grades	Polymerization/recovery scale-up; standard-compliant end-of-life; market demand	Cradle-to-grave advantage and bankable product off-take

## Data Availability

No new data were created or analyzed in this study. Data sharing is not applicable to this article.

## Supplementary Materials

The following supporting information can be downloaded at: https://www.mdpi.com/article/10.3390/polym18202493/s1, Figure S1: Scopus search results used to update the review literature base (2015–2026).

## Abbreviations

The following abbreviations are used in this manuscript

Symbol	Meaning
C5	Five-carbon sugars/pentose sugars
C6	Six-carbon sugars/hexose sugars
Cp	Grain production
Mt	Million tonnes
Mt yr^−1^	Million tonnes per year
wt%	Weight percent
µm	Micrometre
Tg	Glass-transition temperature
SiO_2_	Silicon dioxide/silica
O_2_	Oxygen
CO_2_	Carbon dioxide
H_2_O	Water
